# Malaysian Propolis and Metformin Synergistically Mitigate Kidney Oxidative Stress and Inflammation in Streptozotocin-Induced Diabetic Rats

**DOI:** 10.3390/molecules26113441

**Published:** 2021-06-05

**Authors:** Victor Udo Nna, Ainul Bahiyah Abu Bakar, Zaida Zakaria, Zaidatul Akmal Othman, Nur Asyilla Che Jalil, Mahaneem Mohamed

**Affiliations:** 1 Department of Physiology, Faculty of Basic Medical Sciences, College of Medical Sciences, University of Calabar, P.M.B. 1115 Calabar, Cross River State, Nigeria; victorudon@unical.edu.ng; 2 Department of Physiology, School of Medical Sciences, Universiti Sains Malaysia, Kubang Kerian 16150, Kelantan, Malaysia; ainul@usm.my (A.B.A.B.); zaida_zakaria@student.usm.my (Z.Z.); zaidaakmal@unisza.edu.my (Z.A.O.); 3 Unit of Physiology, Faculty of Medicine, Universiti Sultan Zainal Abidin, Kuala Terengganu 20400, Terengganu, Malaysia; 4 Department of Pathology, School of Medical Sciences, Universiti Sains Malaysia, Kubang Kerian 16150, Kelantan, Malaysia; asyilla@usm.my; 5 Unit of Integrative Medicine, School of Medical Sciences, Universiti Sains Malaysia, Kubang Kerian 16150, Kelantan, Malaysia

**Keywords:** diabetes, inflammation, Malaysian propolis, metformin, nephropathy, oxidative stress

## Abstract

Diabetic nephropathy is reported to occur as a result of the interactions between several pathophysiological disturbances, as well as renal oxidative stress and inflammation. We examined the effect of Malaysian propolis (MP), which has anti-hyperglycemic, antioxidant and anti-inflammatory properties, on diabetes-induced nephropathy. Diabetic rats were either treated with distilled water (diabetic control (DC) group), MP (300 mg/kg b.w./day), metformin (300 mg/kg b.w./day) or MP + metformin for four weeks. We found significant increases in serum creatinine, urea and uric acid levels, decreases in serum sodium and chloride levels, and increase in kidney lactate dehydrogenase activity in DC group. Furthermore, malondialdehyde level increased significantly, while kidney antioxidant enzymes activities, glutathione level and total antioxidant capacity decreased significantly in DC group. Similarly, kidney immunoexpression of nuclear factor kappa B, tumor necrosis factor-*α*, interleukin (IL)-1β and caspase-3 increased significantly, while IL-10 immunoexpression decreased significantly in DC group relative to normal control group. Histopathological observations for DC group corroborated the biochemical data. Intervention with MP, metformin or both significantly mitigated these effects and improved renal function, with the best outcome following the combined therapy. MP attenuates diabetic nephropathy and exhibits combined beneficial effect with metformin.

## 1. Introduction

Diabetic nephropathy (DN) is a common complication reported to occur in approximately 30–40% of diabetic patients, which if not properly managed could result in end-stage renal disease [1]. DN is characterized by increased kidney size and expansion of glomerular volume in early phase of the disease, whereas glomerulosclerosis, tubular atrophy, fibrosis of the interstitium and declined renal function, which presents as increased levels of serum creatinine, urea and uric acid, occur as the disease progress [2]. Studies have shown that DN occurs as a result of hyperglycemia-induced increased reactive oxygen species (ROS) production, which triggers renal oxidative stress, inflammation, apoptosis and fibrosis [1,3]. Animal studies have reported decreased nuclear factor erythroid 2-related factor 2 (Nrf2) protein levels and accompanying decreased antioxidant enzymes activities in the kidney of diabetic rats [2,3]. Furthermore, nuclear factor kappa B (NF-κB) activation, which leads to upregulation of inflammatory cytokines and activation of pro-apoptotic proteins, has also been reported in the kidneys of diabetic rats [4]. The multimechanistic ROS-induced DN necessitates antioxidant supplementation in DM.

The use of natural products in the management of diseases is gaining popularity because of their reported multimechanistic beneficial effects. Propolis, a gluey mixture formed by honeybees from plant materials and their secretions, is reported to have multiple health benefits. In vitro studies on propolis have reported the inhibition of α-amylase and α-glucosidase activities, thus, justifying its anti-hyperglycemic effect [5,6]. Animal and human studies have demonstrated the health benefits of propolis in pathological conditions. Chinese propolis, for example, was reported to improve antioxidant status of diabetic patients after 18 weeks of intervention [7]. In animals, propolis has been reported to have antioxidant, anti-diabetic, anti-inflammatory, anti-apoptotic and anti-bacterial effects [8,9,10,11]. Furthermore, propolis has been reported to protect the reproductive system from diabetes [12,13], heavy metal [14] and chemotherapy-induced [15] impairments.

We previously reported in vitro α-glucosidase inhibition and antioxidant activity of Malaysian propolis (MP) obtained from the stingless bee *Heterotrigona itama* [5]. We also reported that MP decreased blood glucose and increased serum insulin level in diabetic rats, and attributed these effects to decreased pancreatic oxidative stress, inflammation and beta cell apoptosis, and improvement in pancreatic beta cell proliferation [5]. On the basis of our previous report, we designed the present study to assess the effects of MP on DN, which is caused majorly by hyperglycemia-induced increase in ROS production. In recognition of the multifactorial complications of diabetic nephropathy, the present study also investigates the possible synergistic effect of metformin (Met) and MP in halting the progression of diabetic nephropathy at early onset, thus leveraging on the anti-hyperglycemic and antioxidant activities of both therapies.

## 2. Results

### 2.1. Blood Glucose Level

**Figure 1:**
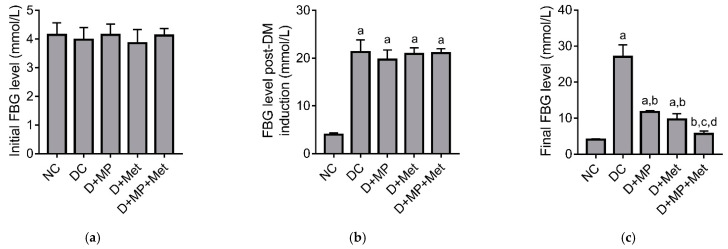
Fasting blood glucose level before (**a**) and after (**b**) STZ injection, and after 4 weeks of treatment with MP, Met or their combination (**c**). NC: normal control, DC: diabetic control, D + MP: diabetic + 300 mg/kg b.w./day of Malaysian propolis, D + Met: diabetic + 300 mg/kg b.w./day of metformin, D + MP + Met: diabetic + Malaysian propolis and metformin combination. Values are mean ± SD, n = 6; ^a^ *p* < 0.05 versus NC; ^b^ *p* < 0.05 versus DC, ^c^ *p* < 0.05 versus D + MP, ^d^ *p* < 0.05 versus D + Met (one-way ANOVA, followed by Tukey post hoc test).

Initial fasting blood glucose (FBG) levels were not significantly different between the groups (Figure 1a). Seventy-two hours post-streptozotocin (STZ) injection, FBG levels increased significantly in all diabetic groups, relative to the normal control (NC) group, indicative of successful diabetes induction (Figure 1b). After four weeks treatment, final FBG levels were significantly higher in diabetic control (DC), diabetic + Malaysian propolis (D+MP) and diabetic + metformin (D + Met) groups, but not in diabetic + Malaysian propolis + metformin (D + MP + Met) group, relative to NC group. Nevertheless, final FBG levels were significantly lower in all treated diabetic groups, relative to DC group. Among the treated diabetic groups, FBG was significantly lower in the combination therapy group, relative to MP and Met monotherapy groups (Figure 1c).

### 2.2. Serum Markers of Renal Function and Renal Lactate Dehydrogenase (LDH) Activity

Serum markers of renal function such as creatinine, urea and uric acid were significantly increased in DC group, relative to NC group, indicative of compromised renal function. Serum creatinine was significantly increased in the monotherapy groups, relative to NC group. Additionally, serum urea level was significantly increased in Met-treated diabetic group, relative to NC group. Nevertheless, serum urea and uric acid levels decreased significantly in all treated diabetic groups, while serum creatinine level decreased significantly only in the combined therapy group, relative to DC group. Among the treated diabetic groups, serum urea level was significantly decreased in D + MP and D + MP + Met groups, compared to D + Met group (Table 1).

Renal LDH activity was significantly increased in DC group, compared to NC group, indicative of tissue damage. Treatment with MP, Met or both, significantly decreased renal LDH activity, with the combination therapy having the best result, and significantly lower compared to D + Met group (Table 1).

### 2.3. Serum Electrolytes

We found significant decreases in serum sodium and chloride levels, but significant increase in potassium level in DC group, relative to NC group (Table 1). Treatment with MP, Met or both, significantly increased serum sodium and chloride levels, and decreased potassium level, relative to DC group. However, sodium and chloride levels were significantly decreased in D + Met group, relative to NC group, while chloride level was significantly increased in D + Met group relative to D + MP and D + MP + Met groups (Table 1).

### 2.4. Kidney Histopathology

**Figure 2:**
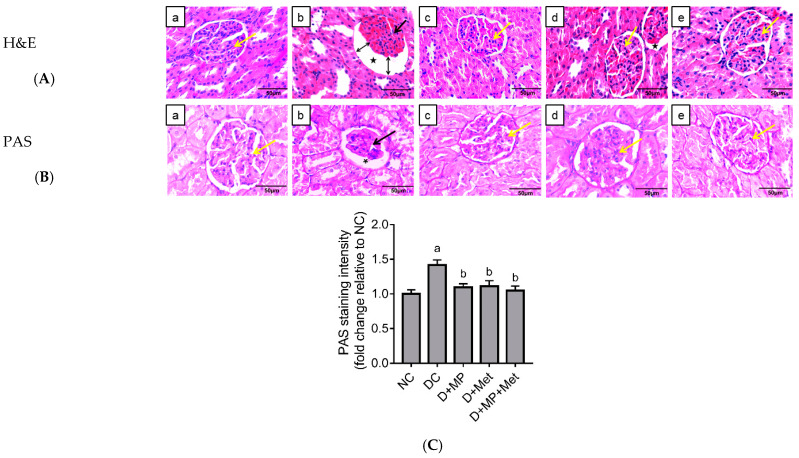
Representative photomicrographs of the histology of the kidney (H & E and PAS) of NC (a), DC (b), D + MP (c), D + Met (d) and D + MP + Met (e) groups. H & E staining of the renal cortex (**A**) in DC group showed collapsed glomerulus tuft (black arrow) and large urinary/Bowman’s space (*), while PAS staining (**B**) showed glomerulosclerosis (black arrow), with mild atrophy of renal tubules in the renal medulla. The treated diabetic groups (c, d and e), especially D + MP + Met (e), showed near normal glomerulus structures (yellow arrow) with no collapse of glomerular tuft or glomerulosclerosis and marked improvement of tubular atrophy, comparable with NC group (a). Photographs were taken using a 40× objective (scale bar: 50 µm). For PAS staining intensity (**C**), values are mean ± SD, n = 6; ^a^ *p* < 0.05 versus NC; ^b^ *p* < 0.05 versus DC (one-way ANOVA, followed by Tukey post hoc test).

Kidney sections were stained with hematoxylin and eosin (H & E) and periodic acid–Schiff (PAS) stains (Figure 2a–c). H & E section of the kidney in the NC group (a) showed normal histological structures of the glomeruli, renal tubules and interstitium, whereas in DC group (b), collapsed/reduced glomeruli tuft and increased Bowman’s space with an area of sclerosis were noted, as highlighted by the significant increase in PAS stain (Figure 2b,c). Mild tubular atrophy was also seen. In D + MP group (c), near normal glomeruli and renal tubules were seen, which was comparable to NC group. In D + Met group (d), compared to the NC group, no collapse of the glomerular tuft was seen. Only minimal mesangial matrix deposition/sclerosis was observed, as highlighted by the significant decrease in PAS stain (Figure 2b,c). However, in D + MP + Met group (e), there was restoration to near normal appearance as there was marked improvement noted on the glomerular structures and tubules of the kidney.

### 2.5. Renal Antioxidant/Oxidant Status

**Figure 3:**
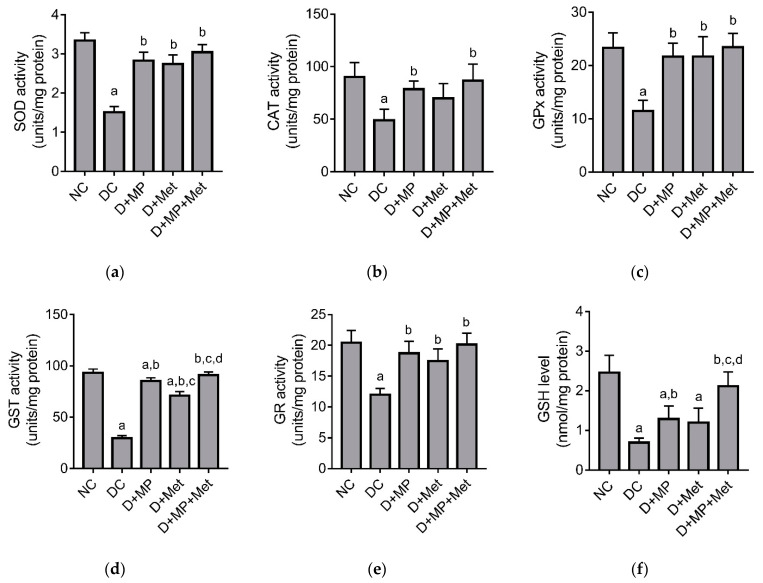
Effect of MP, Met and their combination on (**a**) SOD, (**b**) CAT, (**c**) GPx, (**d**) GST and (**e**) GR activities, and (**f**) GSH level in the kidney of diabetic rats. NC: normal control, DC: diabetic control, D + MP: diabetic + 300 mg/kg b.w./day of Malaysian propolis, D + Met: diabetic + 300 mg/kg b.w./day of metformin, D + MP + Met: diabetic + Malaysian propolis and metformin combination. SOD: superoxide dismutase; CAT: catalase; GPx: glutathione peroxidase; GR: glutathione reductase; GST: glutathione-S-transferase; GSH: total glutathione. Values are mean ± SD, n = 6; ^a^ *p* < 0.05 versus NC; ^b^ *p* < 0.05 versus DC, ^c^ *p* < 0.05 versus D + MP, ^d^ *p* < 0.05 versus D + Met (one-way ANOVA, followed by Tukey post hoc test).

Oxidative stress was evident in the kidneys of DC group, as seen with significant decreases in superoxide dismutase (SOD), catalase (CAT), glutathione peroxidase (GPx), glutathione-s-transferase (GST) and glutathione reductase (GR) activities, and glutathione (GSH) level in DC group, relative to NC group (Figure 3). We also observed significant decreases in SOD and CAT activities, and GSH level in D+MP and D + Met groups, relative to NC group. Interestingly, apart from CAT activity and GSH level in D + Met group, we found significant increases in antioxidant enzyme activities and GSH level in all treated diabetic groups, relative to DC group (Figure 3). Among the treated diabetic groups, GST activity was significantly decreased in D + Met group, relative to D+MP and D + MP + Met groups, and significantly increased in D + MP + Met group relative to D+MP group. Additionally, GSH level was significantly increased in D + MP + Met group, relative to D+MP and D + Met groups (Figure 3).

**Figure 4:**
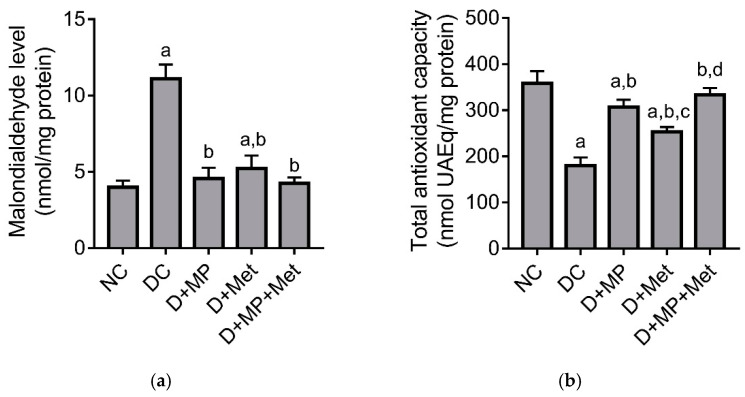
Effect of MP, Met and their combination on (**a**) malondialdehyde level and (**b**) total antioxidant capacity in the kidney of diabetic rats. NC: normal control, DC: diabetic control, D + MP: diabetic + 300 mg/kg b.w./day of Malaysian propolis, D + Met: diabetic + 300 mg/kg b.w./day of metformin, D + MP + Met: diabetic + Malaysian propolis and metformin combination. Values are mean ± SD, n = 6; ^a^ *p* < 0.05 versus NC; ^b^ *p* < 0.05 versus DC, ^c^ *p* < 0.05 versus D + MP, ^d^ *p* < 0.05 versus D + Met (one-way ANOVA, followed by Tukey post hoc test).

Malondialdehyde, a marker of lipid peroxidation, increased significantly, while total antioxidant capacity (TAC) decreased significantly in DC group, compared to NC group. In addition, MDA level increased significantly in D + Met group, while TAC decreased significantly in D+MP and D + Met groups, compared to NC group (Figure 4). Nevertheless, MDA level decreased significantly, and TAC increased significantly in all the treated diabetic groups compared to DC group. Among the treated groups, MDA level did not significantly differ, but TAC was significantly decreased in D + Met group, relative to D+MP and D + MP + Met groups (Figure 4).

### 2.6. Immunoexpression of Inflammatory Markers in the Kidney

**Figure 5:**
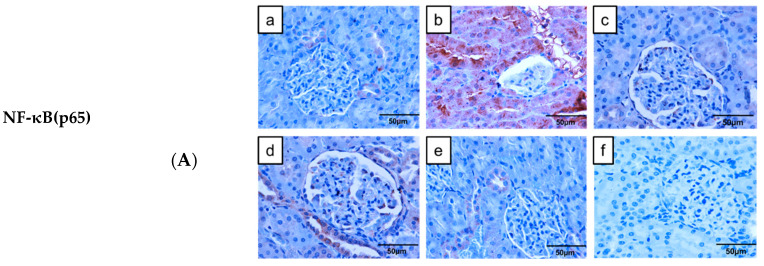

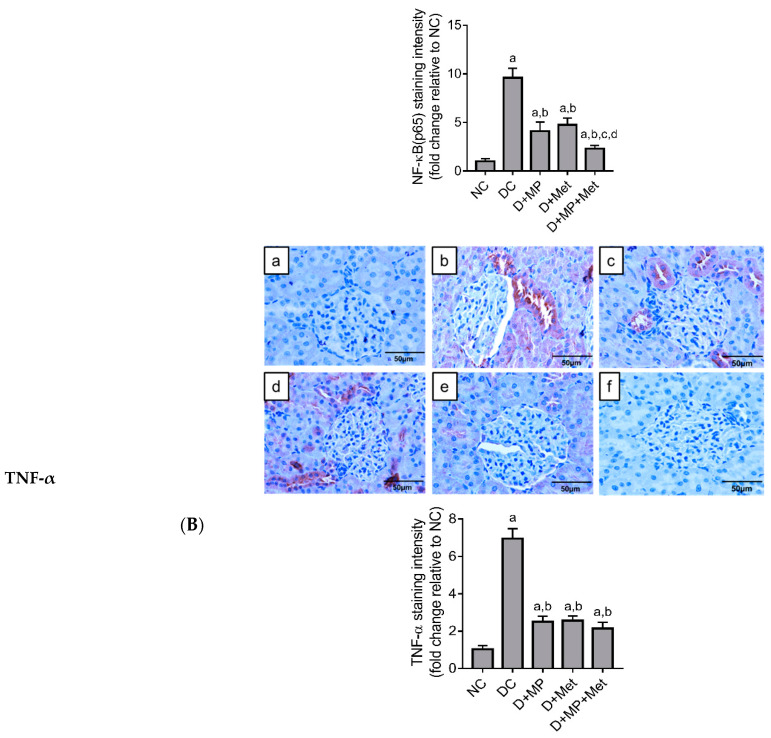
Effect of MP, immunoexpressions of NF-κB(p65) (**A**) and TNF-α (**B**) in the kidney of diabetic rats. (a) NC, (b) DC, (c) D + MP, (d) D + Met and (e) D + MP + Met groups. Negative control is presented as (f). Photographs were taken at 400× magnification (scale bar = 50 µm). For quantitative data, 5 photographs were analyzed per slide using ImageJ software. The expression levels were expressed as fold change relative to NC group. Values are mean ± SD, n = 6; ^a^ *p* < 0.05 versus NC; ^b^ *p* < 0.05 versus DC, ^c^ *p* < 0.05 versus D + MP, ^d^ *p* < 0.05 versus D + Met (one-way ANOVA, followed by Tukey post hoc test).

**Figure 6:**
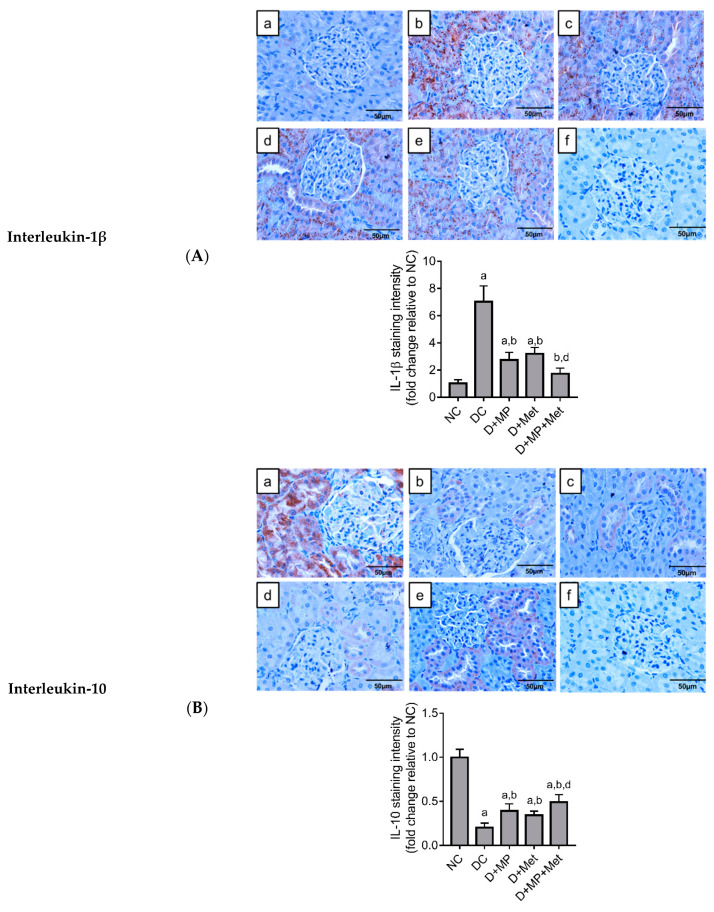
Immunoexpressions of interleukin-1β (**A**) and interleukin-10 (**B**) in the kidney of diabetic rats. (a) NC, (b) DC, (c) D + MP, (d) D + Met and (e) D + MP + Met groups. Negative control is presented as (f). Photographs were taken at 400× magnification (scale bar = 50 µm). For quantitative data, 5 photographs were analyzed per slide using ImageJ software. The expression levels were expressed as fold change relative to NC group. Values are mean ± SD, n = 6; ^a^ *p* < 0.05 versus NC; ^b^ *p* < 0.05 versus DC, ^d^ *p* < 0.05 versus D + Met (one-way ANOVA, followed by Tukey post hoc test).

We examined selected markers that promote inflammatory response, notably NF-κB(p65), tumor necrosis factor (TNF)-*α* and interleukin (IL)-1β, and IL-10 (anti-inflammatory marker) using immunohistochemical technique, and found significant fold increases in NF-κB (p65) (Figure 5a), TNF-*α* (Figure 5b) and IL-1β (Figure 6a) expressions, and significant decrease in IL-10 expression (Figure 6b) in kidney sections of DC group, relative to NC group.

Although the expressions of NF-κB(p65), TNF-*α* and IL-1β were significantly increased and IL-10 expression significantly decreased in all treated diabetic groups, relative to NC group, we found significant decreases in NF-κB(p65), TNF-*α* and IL-1β expressions and significant increase in IL-10 expression in all treated diabetic groups, compared to DC group. Among the treated diabetic groups, NF-κB(p65) expression was significantly decreased in D + MP + Met group, relative to D + MP and D + Met groups. Additionally, IL-1β expression decreased significantly, while IL-10 increased significantly in D + MP + Met group, relative to D + Met group (Figure 6b).

### 2.7. Immunoexpression of Cleaved Caspase-3 in the Kidney

**Figure 7:**
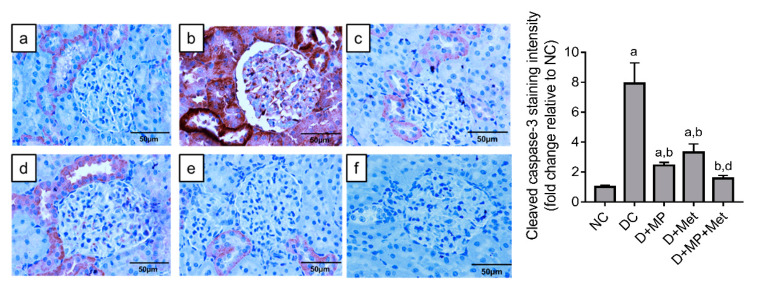
Cleaved caspase-3 immunoexpression in the kidney of diabetic rats. (**a**) NC, (**b**) DC, (**c**) D + MP, (**d**) D + Met and (**e**) D + MP + Met groups. Negative control is presented as (**f**). Photographs were taken at 400× magnification (scale bar = 50 µm). Immunoexpression of cleaved caspase-3 was presented as fold change relative to NC group. Values are mean ± SD, n = 6; ^a^ *p* < 0.05 versus NC; ^b^ *p* < 0.05 versus DC, ^d^ *p* < 0.05 versus D + Met (one-way ANOVA, followed by Tukey post hoc test).

We found significant increase in cleaved caspase-3 expression in the kidney of DC, D + MP and D + Met groups, relative to NC group. However, cleaved caspase-3 expression decreased significantly in all treated diabetic groups, relative to DC group, with D + MP + Met group significantly decreased compared to D + Met group (Figure 7).

## 3. Discussion

Diabetic nephropathy is a common complication of DM, which may result in chronic renal failure and/or end-stage renal disease in most of the cases, if not properly managed [16]. Since most of the complications associated with DM (including DN) are associated with oxidative stress, we examined the effect of treating diabetic rats with MP, which we previously reported to have significant anti-hyperglycemic, antioxidant, anti-inflammatory and anti-apoptotic properties [5,10], on kidney function.

Increases in serum creatinine, urea and uric acid levels are considered as markers of nephropathy. Consistent with previously reported findings [3,16], we found significant increases in serum creatinine, urea and uric acid, as well as decreases in sodium and chloride levels, indicative of nephropathy. Additionally, we observed a significant increase in LDH activity in the kidney of untreated diabetic rats, thus strengthening our hypothesized renal damage. Increased LDH activity has been reported in incidences of tissue damage, thus serving as a biomarker for tissue damage [17]. The functional damage discussed above was accompanied by structural damage, as shown by the histological features. We observed increased Bowman’s space, atrophied glomerular tuft in numerous Bowman’s capsules, and intense glomerular staining with PAS, suggestive of early signs of glomerulosclerosis. In fact, previous research reported increases in collagen IV and hydroxyproline levels in the kidney of diabetic rats after four weeks of DM induction, indicative of early renal fibrosis [16]. This supports our hypothesis of structural damage in the kidney of untreated diabetic rats in the present study. The observed histological findings justify the increases in serum creatinine, urea and uric acid levels. Treatment with MP and metformin separately and in combination significantly restored both functional and structural integrity of the kidney in the present study. Our results are consistent with previously reported findings, which showed improved renal function in diabetic rats treated with propolis from Saudi Arabia [9], China and Brazil [18].

Research has shown that metabolic abnormalities, which are characteristic of DM, trigger the overproduction of mitochondrial superoxide, leading to oxidant/antioxidant imbalance, resulting in oxidative stress [19]. Furthermore, the emergence of oxidative stress has been reported to precede diabetic complications [19]. Oxidative stress was evident in the kidney of untreated diabetic rats in the present study, as we found significant decrease in total antioxidant capacity and increase in MDA level in the kidney of untreated diabetic rats. These were accompanied by significant decreases in GSH levels and activities of antioxidant enzymes, notably SOD, CAT, GPx, GST and GR, which are consistent with previous studies [3]. Interestingly, MP seems to have decreased renal oxidative stress in the present study, since it decreased MDA level, increased antioxidant enzymes activities and increased TAC. The significantly higher TAC in MP-treated diabetic rats relative to metformin-treated diabetic rats suggests that MP has antioxidant properties besides its anti-hyperglycemic effect. Metformin may have improved TAC in the kidneys indirectly through its effect on glycemic control, while MP may have improved TAC in the kidneys through indirect and direct mechanisms. We hypothesize that MP’s indirect mechanism involves modulation of blood glucose, thus decreasing ROS production. This hypothesis is supported by our previous experiments that showed that MP inhibited *α*-glucosidase in vitro and improved pancreatic *β*-cell function, which culminated into increased serum insulin and decreased blood glucose level [5]. On the other hand, the direct mechanism involves scavenging free radicals, thus complementing endogenous antioxidants, and likely activating Nrf2, which is known to stimulate antioxidant gene expression [20,21]. Our previous experiments showed that MP, which contained large amounts of phenols and flavonoids, significantly inhibited DPPH radical, had ferric ion reducing activity and H_2_O_2_ scavenging activity [5]. This supports our hypothesized direct effect of MP on oxidants in the kidney of treated diabetic rats. Our findings are consistent with previous reports, which showed improved kidney antioxidant status in diabetic rats after treatment with propolis from China, Brazil and Saudi Arabia [9,18].

Indeed, natural products have been reported to activate Nrf2 as part of their mechanism in increasing antioxidant enzyme activity when administered in disease state. This effect has been attributed to the rich flavonoid and phenolic compounds in natural products [3,22]. For example, research has shown that resveratrol, quercetin and gallic acid upregulate Nrf2 and the downstream expression of antioxidant genes in various disease conditions [23,24,25,26]. Interestingly, our group identified the presence of coumaric acid derivatives, ellagic acid, gallic acid derivatives and resveratrol in MP using LC–MS analysis [27], which might explain the observed improvement in TAC in the kidney of treated diabetic rats in the present study.

The link between persistent hyperglycemia, oxidative stress, inflammation and apoptosis, has been long established. Increased ROS production as observed in DM, activates inflammatory response, which exacerbates the deleterious effects of the already existing oxidative stress, thus creating a viscous cycle of events that culminate in apoptotic cellular damage [26]. In the present study, the immunoexpression of NF-κB increased in the kidney of untreated diabetic rats. NF-κB is a transcription factor, which upregulates the expression of inflammatory cytokines. As observed in the present study, increased NF-κB expression was accompanied by an increase in TNF-α and IL-1β expressions, and a decrease in IL-10, which is anti-inflammatory, suggestive of the involvement of inflammation in the pathogenesis of nephropathy in the untreated diabetic rats. Previous studies reported NF-κB activation and increase in pro-inflammatory (iNOS, TNF-*α*, IL-1β and IL-6) and pro-apoptotic (Bax, caspase-9 and caspase-3) proteins expression in diabetic rat kidneys [3,4]. Consistent with these studies, we found significant increase in cleaved caspase-3 immunoexpression in the kidney of untreated diabetic rats, suggestive of apoptosis. Interestingly, MP decreased immunoexpression of the pro-inflammatory markers and cleaved caspase-3 in the present study. This may be attributable to the antioxidant, anti-inflammatory and anti-apoptotic effects of MP. We previously demonstrated that MP decreased NF-κB, TNF-*α*, IL-1β and cleaved caspase-3 immunoexpressions, and increased IL-10 and proliferating cell nuclear antigen immunoexpressions in the pancreas, liver and testes of diabetic rats [5,10,12]. In the present study, metformin’s beneficial effect in attenuating inflammation and apoptosis in the kidneys of diabetic rats may be attributed to its effect on glycemic control as discussed earlier. The combined therapy proved to be more effective in ameliorating inflammation and apoptosis in the present study. To the best of our knowledge, our study is the first to report the beneficial effects of Malaysian propolis on renal functions of diabetic rats. Although not all the possible mechanisms of MP’s beneficial effects were exhausted in the present study, we created a lead for future research to potential mechanisms in the present study.

## 4. Materials and Methods

### 4.1. Chemicals

Rabbit polyclonal primary antibodies for caspase-3 (PAA626Ra01), NF-κB (PAB824Ra01), TNF-α (PAA133Ra01), IL-1β (PAA563Ra01) and IL-10 (PAA056Ra01) were purchased from Cloud-Clone Corp, Katy, TX, USA. Dako DAB+ substrate chromogen (K3468) and Dako EnVision™+ System/HRP (K4003) were purchased from Agilent Technologies, Inc., USA. Metformin was purchased from Hovid Bhd, Malaysia, while STZ was purchased from Sigma-Aldrich (USA). All other reagents were of analytical grade.

### 4.2. Preparation of Propolis Ethanol Extract

We purchased Malaysian propolis *itama* from a local bee keeper in Min House Camp, Kelantan, Malaysia. MP was extracted using 70% ethanol following our previously described protocol [5]. The freeze-dried propolis extract was kept at −20 °C until usage. In our previous investigation, we reported antioxidant activity and α-glucosidase inhibition activity of MP extract, in addition to the presence of large amounts of flavonoid and phenolic compounds [5].

### 4.3. Experimental Animals

Thirty adult male Sprague Dawley rats weighing 250–300 g were employed for this study. They were obtained from the Animal Research and Service Centre, Universiti Sains Malaysia (USM), Kelantan, Malaysia, and housed in the Department of Physiology animal room. The rats were maintained in 12/12 h light/dark cycle and had ad libitum access to standard pellet diet and drinking water. They were acclimatized for 7 days before the experiment. The current study was approved by the animal ethics committee of USM, with approval number USM/IACUC/2017/(831). The rats were maintained in accordance with the Guidelines for the Care and Use of Laboratory Animals, National Institute of Health.

### 4.4. Study Design

The rats were randomly assigned to five groups (n = 6/group): normal control (NC), diabetic control (DC), diabetic + 300 mg/kg b.w./day of MP (D+MP), diabetic + 300 mg/kg b.w./day of metformin (D + Met) and diabetic + MP + Met (D + MP + Met). The doses for MP and metformin were as used in our previous study [5]. The rats were treated for 4 weeks, after which they were sacrificed under pentobarbital (60 mg/kg b.w.) anesthesia after an overnight fast.

### 4.5. Induction of Diabetes Mellitus

Diabetes mellitus was induced using a single intraperitoneal injection of STZ (60 mg/kg b.w.). STZ was dissolved in ice-cold saline and administered after an overnight fast. The rats were given ad libitum access to 5% glucose which they drank overnight, post-STZ injection, to prevent hypoglycemia and mortality. Rats with fasting blood glucose ≥ 14 mmol/L, as recorded using a glucometer (URight TD-4279 Blood Glucose Monitoring System, Dusseldorf, Germany) 72 h post-STZ injection, were considered diabetic and employed for the present study.

### 4.6. Measurement of Serum Biochemical Markers of Renal Function

To assess serum markers of renal function, blood was collected into plain tubes via cardiac puncture and allowed to stand for 2 h before centrifuging at 4000 rpm for 15 min to collect serum. The serum obtained was used for determining creatinine, urea, uric acid, sodium, chloride and potassium levels by using an Architect ci8200 Integrated system (Abbott, IL, USA) [28].

### 4.7. Histopathological Assessment

The left kidney was used for histopathological study. Briefly, the kidney was fixed in 10% formalin for 48 h, excised and processed with an automated tissue processor (Leica, Nussloch, Germany), before embedding in blocks of paraffin. Four µm thick sections were used for H & E and PAS staining. Thereafter, the sections were observed under a light microscope (Olympus BX41, Tokyo, Japan for assessment on the presence of glomerulosclerosis and thickening of the medullary collecting ducts at 400× magnification. PAS-stained sections were analyzed using ImageJ software (ImageJ, NIH-Bethesda, MD, USA) for staining intensity.

### 4.8. Assessment of Renal Tissue Oxidative Stress Status

#### 4.8.1. Preparation of Kidney Tissue Homogenate

The right kidney was excised and rinsed in 0.1 M Tris-HCl buffer (pH 7.4). Ten percent (10%) tissue homogenate was prepared in the same buffer and centrifuged at 1000× *g* for 20 min using a refrigerated centrifuge (Eppendorf, Taufkirchen, Germany). The supernatant was collected for determination of SOD, CAT, GPx, GST and GR activities, GSH and malondialdehyde levels, and TAC. The results were normalized with protein concentrations of each sample as determined using a commercially available protein assay kit (BioAssay Systems, Hayward, CA, USA). Kidney LDH activity was determined using a commercially available LDH colorimetric assay kit (BioAssay Systems, Hayward, CA, USA).

#### 4.8.2. Determination of Antioxidant Enzymes Activities and Total Glutathione Level

The activity of SOD was assessed using nitro tetrazolium blue reduction method as previously described [29]. The activity of CAT was assessed using hydrogen peroxide substrate, which forms a yellowish complex with molybdate upon decomposition [30]. The activity of GPx was assessed following a method that is based on glutathione oxidation by hydrogen peroxide [31]. The activity of GR was determined using a method that is based on glutathione disulfide reduction in the presence of NADPH, which is later oxidized to NADP^+^ [32]. The activity of GST was assessed using a method based on GSH conjugation with 1-chloro-2,4-dinitrobenzene as substrate [33]. Total GSH level was determined using a method based on 5-thio-2-nitrobenzoic acid reduction. In this assay, 5,5-dithiobis-2-nitrobenzoic acid reacts with the sulfhydryl group of GSH to form 5-thio-2-nitrobenzoic acid [34].

#### 4.8.3. Determination of Malondialdehyde Level and Total Antioxidant Capacity

Kidney homogenate malondialdehyde level was measured using a previously described method, with tetra ethoxy propane as standard [35], while total antioxidant capacity was measured using a method that is based on reduction in thiobarbituric acid reactive substance formation [36].

### 4.9. Immunohistochemical Detection of Inflammatory Markers and Cleaved Caspase-3

Antigen retrieval was performed on 4 µm thick sections using a pressure cooker containing Tris-EDTA buffer with 0.05% Tween 20 (pH 9.0) for 3 min. Thereafter, endogenous peroxidase was blocked by incubating the sections with 3% hydrogen peroxide in PBS for 5 min, followed by washing with distilled water and Tris-buffered saline containing 0.05% Tween 20 (TBST, pH 8.4). Incubation with polyclonal primary antibodies for NF-κB (1:80), TNF-α (1:120), IL-1β (1:80), IL-10 (1:40) and caspase-3 (1:150) was done at 4 °C overnight. After washing twice with TBST, sections were incubated with Dako EnVision™+ System/HRP labeled polymer containing goat anti-rabbit secondary antibody (Agilent Technologies, Inc., Santa Clara, CA, USA) for 30 min at room temperature. Visualization was performed using Dako 3,3′-diaminobenzidine substrate (Agilent Technologies, Inc., Santa Clara, CA, USA) at room temperature for 5 min. Thereafter, the sections were counterstained for 5 sec with hematoxylin, dehydrated and examined under a light microscope (Olympus BX41, Tokyo, Japan). The brown staining intensity was determined using ImageJ software (ImageJ, NIH-Bethesda, MD, USA).

### 4.10. Statistical Analysis

Data obtained in this study are presented as mean ± standard deviation (SD). One-way analysis of variance (ANOVA) was used, followed by Tukey post hoc test to determine differences between groups. GraphPad Prism 7.0 (GraphPad Software Inc., La Jolla, CA, USA) was used for the analysis, and *p* values < 0.05 were considered statistically significant.

## 5. Conclusions

Malaysian propolis attenuated renal oxidative stress and inflammation in diabetic rats in the present study. We hypothesize that these beneficial effects are responsible for the improved renal function as shown by decreases in serum creatinine, urea and uric acid levels in the treated diabetic rats, besides the glucose-lowering effect. Additionally, we found that its beneficial effects were potentiated by metformin, thus making MP a potential complementary therapy in DN. The more potent effect of the complementary therapy over the monotherapies may be associated with the rich polyphenol content of MP, since polyphenols are known to show a myriad of biological activities.

## Figures and Tables

**Table 1:** Effect of MP, metformin or their combination, on serum renal function markers and kidney LDH activity in all the groups.

Parameters	NC	DC	D + MP	D + Met	D + MP + Met
Creatinine (µmol/L)	45.75 ± 1.46	54.10 ± 1.73 ^a^	50.77 ± 1.05 ^a^	50.00 ± 4.81 ^a^	48.55 ± 0.68 ^b^
Urea (mmol/L)	7.93 ± 0.93	25.35 ± 1.62 ^a^	6.67 ± 2.16 ^b^	15.00 ± 4.07 ^a,b,c^	5.15 ± 0.76 ^b,d^
Uric acid (µmol/L)	82.72 ± 8.79	133.50 ± 17.62 ^a^	83.72 ± 19.64 ^b^	73.77 ± 13.46 ^b^	90.68 ± 14.38 ^b^
Sodium (mmol/L)	145.20 ± 1.47	134.00 ± 1.27 ^a^	142.50 ± 3.02 ^b^	140.80 ± 2.04 ^a,b^	142.50 ± 0.84 ^b^
Chloride (mmol/L)	100.20 ± 0.75	91.83 ± 0.75 ^a^	99.00 ± 4.86 ^b^	94.17 ± 2.23 ^a,d^	99.33 ± 0.82 ^b,d^
Potassium (mmol/L)	5.22 ± 0.19	6.97 ± 0.92 ^a^	5.40 ± 0.26 ^b^	5.42 ± 0.33 ^b^	5.08 ± 0.27 ^b^
LDH (IU/g tissue)	426.70 ± 42.22	736.50 ± 97.85 ^a^	445.30 ± 50.68 ^b^	526.30 ± 38.42 ^b^	388.40 ± 45.06 ^b,d^

LDH: lactate dehydrogenase, NC: normal control, DC: diabetic control, D + MP: diabetic + 300 mg/kg b.w./day of Malaysian propolis, D + Met: diabetic + 300 mg/kg b.w./day of metformin, D + MP + Met: diabetic + Malaysian propolis + metformin combination. Values are mean ± SD, n = 6; ^a^ *p* < 0.05 vs NC; ^b^ *p* < 0.05 versus DC, ^c^ *p* < 0.05 versus D + MP, ^d^ *p* < 0.05 versus D + Met (one-way ANOVA, followed by Tukey post hoc test).

## Data Availability

The datasets for this manuscript can be obtained from the corresponding author upon reasonable request.

## Sample Availability

Samples of the product are available from the authors.
